# Enhanced Chroma-YOLO Framework for Effective Defect Detection and Fatigue Life Prediction in 3D-Printed Polylactic Acid

**DOI:** 10.3390/ma18225159

**Published:** 2025-11-13

**Authors:** Liang Wang, Zhibing Liu, Ting Lv, Xibin Wang, Tianyang Qiu

**Affiliations:** 1 School of Mechanical Engineering, Beijing Institute of Technology, No.5 Zhongguancun South Street, Haidian District, Beijing 100081, China; 2 Chongqing Tiema Inductries Group Co., Ltd., No.43 Yangjiaping Main Street, Jiulongpo District, Chongqing 400050, China

**Keywords:** polylactic acid, defect detection, random forest, fatigue life prediction

## Abstract

Internal defects commonly occur during the 3D printing process of Polylactic Acid (PLA), and significant challenges remain in detecting and extracting these defects, as well as understanding the relationship between defects and material fatigue life. This research proposes the Chroma-YOLO Enhanced Integrated Framework, an improved YOLOv11n-based model that integrates HSV defect extraction module and a random forest prediction model. Comprehensive ablation experiments demonstrate that the Chroma-YOLO model achieves significant improvements of 6.9% and 7.3% for mAP50 and mAP50-95 metrics, respectively, compared to the baseline YOLOv11n model, confirming substantial enhancements in feature extraction capability and target localization accuracy. Furthermore, this framework establishes a comprehensive model from defect detection to fatigue life prediction by combining the HSV color space-based defect detection technique with the random forest machine learning algorithm. The random forest-based predictive model achieves a remarkable accuracy of 96.25% and 99.09%for the test and validation set, respectively, for fatigue life prediction of 3D-printed PLA, which shows significant improvement compared to the conventional prediction methodologies.

## 1. Introduction

Fused Deposition Modeling (FDM), a widely Additive Manufacturing (AM) process [1], has seen rapid expansion in applications over the past decade [2]. Polylactic acid (PLA), known for its excellent biodegradability and environmentally friendly characteristics, has become one of the most widely used materials in FDM, particularly in biomedical implant manufacturing [3]. However, PLA implants typically endure complex varying loads during service in the human body [4], which can result in fatigue failure [5]. Thus, accurate assessment of the fatigue behavior of PLA implants under varying loading conditions has significant clinical implications [6].

The random distribution of internal defects in additively manufactured specimens significantly impacts the consistency of material mechanical properties, thereby exerting a decisive influence on fatigue life. X-ray computed tomography (XCT), a high-resolution non-destructive testing technology, has been widely applied in defect analysis of additively manufactured parts [7]. However, XCT has limitations in detecting polymer materials, including low image contrast, noise interference, and limited spatial resolution, rendering traditional image analysis methods inadequate for identifying internal defects with low contrast and blurred boundaries [8]. In recent years, deep learning technology has achieved breakthrough progress in the field of additive manufacturing defect detection. Bellens et al. improved XCT image segmentation of polymer AM parts through U-net segmentation algorithms [9]; Gobert et al. developed the ACTS tool for automated segmentation of pores in XCT images of metal AM parts [10]; Zhang et al. proposed a lattice structure defect detection method based on improved YOLOv3 [11]. In research correlating defects with fatigue performance, Snow et al. evaluated the fatigue performance of AM parts by combining XCT data with CNN models [12]; Luo et al. analyzed the relationship between porosity characteristics and fatigue life [13]; Li et al. proposed a fatigue life prediction method based on multi-modal transfer learning [14].

Deep learning, an algorithmic system with multi-layered neural networks, automatically learns implicit feature structures in data through supervised, semi-supervised, or unsupervised methods [15] and transforms image pixel data into higher-order, abstract feature representations [16]. This advantage gives deep learning unique value in object detection tasks within the computer vision field, enabling precise identification and localization of objects in images or videos [17]. Deep learning-based object detection algorithms are primarily categorized into two technical paradigms: two-stage and one-stage methods, each with distinct characteristics and suited to different application scenarios [18].

Two-stage algorithms consist of localization and classification steps. The first generates candidate regions, and the second classifies objects in those regions. Despite their improved accuracy, two-stage detectors are generally slower. Algorithms in this category include R-CNN [19], Fast R-CNN [19], and Faster R-CNN [20]. One-stage detection algorithms represent a significant methodology in object detection, directly predicting object positions and categories from input images without additional region proposal steps. These algorithms simultaneously accomplish object localization and classification tasks through a single network structure, offering advantages of fast processing and strong real-time performance. Typical one-stage detection algorithms include the YOLO series, RetinaNet [21], and Single Shot MultiBox Detector (SSD) [22]. YOLO provides fast detection and high localization precision, which are essential for identifying complex internal defects in 3D-printed PLA. We also note that traditional image segmentation or simple CNN methods show limitations in detecting small or low-contrast defects.

The rapid progress of computer vision through deep learning has led to the widespread adoption of YOLO (You Only Look Once) models in defect detection, owing to their ability to process in real-time and their efficiency. YOLO model applications in defect detection face three main challenges: insufficient multi-scale feature fusion, lack of attention mechanisms, and low localization accuracy for irregularly shaped objects. To address these issues, Wang et al. improved the YOLOv11 model by introducing SimAM attention modules, DySample upsampling modules, and Shape-IoU loss functions, significantly enhancing the model’s detection accuracy and robustness in complex scenarios [23]. Zhao et al. proposed RDD-YOLO, an improved steel surface defect detection model based on YOLOv5, which significantly enhanced detection accuracy and speed through backbone network improvements and CIoU loss function optimization [24]. Lu et al. developed a real-time defect detection system based on YOLOv3, which performed well in additive manufacturing of continuous fiber-reinforced polymer composites but struggled to identify small defects [9]. Wu et al.’s improved YOLOv3 algorithm encountered difficulties in feature extraction and detecting small targets during online wire arc additive manufacturing defect detection [25]. Wen et al. proposed an improved YOLOv7-based method for detecting complex internal defects in 3D printed lattice structures. By integrating CBAM and ASFF, this method significantly improved detection accuracy and multi-scale adaptability, providing an efficient solution for industrial non-destructive testing [26]. Research by Sani et al. demonstrated that although YOLOv11 outperformed other YOLO versions in FDM printing defect detection, it still exhibited high miss rates in low-contrast environments [27]. Despite these successes, existing methods still face limitations in feature discrimination and flexibility of multi-scale fusion strategies when addressing low-contrast and multi-morphological defects in internal polylactic acid specimens. Internal defect detection in polylactic acid specimens faces several challenges. Traditional object detection networks often lack sufficient feature extraction capability in complex background environments, making it difficult to accurately identify multi-scale and multi-morphological defects simultaneously. Conventional RGB-based methods have limited effectiveness for detecting low-contrast and morphologically complex internal defects. More critically, there has long been a lack of effective connection between defect detection and material performance evaluation, with no direct mapping mechanism established from defect characteristics to fatigue life.

This research presents the Chroma-YOLO Enhanced Integrated Framework to tackle these challenges, an improved YOLOv11n-based model that integrates HSV defect extraction and a random forest prediction algorithm, featuring five core innovations: (1) The AttForm feature extraction module that combines depth-separable convolution with multi-head self-attention mechanisms through a gated structure, simultaneously improving feature extraction efficiency and the ability to focus on key regions, effectively addressing the inadequacies of traditional convolution in balancing computational complexity and precision, as well as the problem of attention dispersion in complex backgrounds; (2) The TriFusion feature fusion architecture that achieves dynamic adaptive integration of high, medium, and low-level features through an EdgeMixer edge attention fusion mechanism, resolving the limitations of static fusion strategies in traditional feature pyramid networks; (3) The RepFocus reparametrized self-attention network that significantly improves model efficiency during the inference phase through reparametrized design of attention mechanisms, while maintaining high-precision representation capabilities during training; (4) The BoundaryIoU (BIOU) boundary penalty loss function that precisely adjusts bounding box positions through a boundary distance penalty mechanism, solving the problem of insufficient precision in traditional IoU loss functions when processing small targets and irregularly shaped defects; (5) An innovative integration of HSV color space enhancement technology with random forest prediction models that both breaks through the limitations of traditional RGB and XCT imaging in identifying low-contrast defects and establishes a direct mapping relationship between defect features and material life through key defect parameters, effectively resolving the technical bottleneck of disconnection between defect detection and performance evaluation.

This study aims to develop an integrated approach for detecting internal defects and predicting the fatigue life of 3D-printed PLA materials by combining HSV-based defect extraction with an improved YOLO model. A Random Forest algorithm is then employed to establish the relationship between defect features and fatigue behavior. The central hypothesis is that fatigue life can be quantitatively predicted from defect area features extracted from CT slice images. The detected defect types include pores, cracks, and interlayer separations, and the analysis was performed on two-dimensional CT slices. The remainder of this paper is structured as follows: Section 2 introduces the experimental methods; Section 3 describes the experiments and results; Section 4 presents the conclusions of this paper.

## 2. Experimental Methods

### 2.1. Internal Defect Characterization and Fatigue Testing of PLA Specimens

The specimens were made from 4032D NatureWorks polylactic acid. Eighteen specimens were printed using identical parameters on a fused deposition modeling (FDM) 3D printer. Printing parameters were set as follows: Printing temperature 180 °C, layer height 0.1 mm, and infill density 10%.

**Figure 1:**
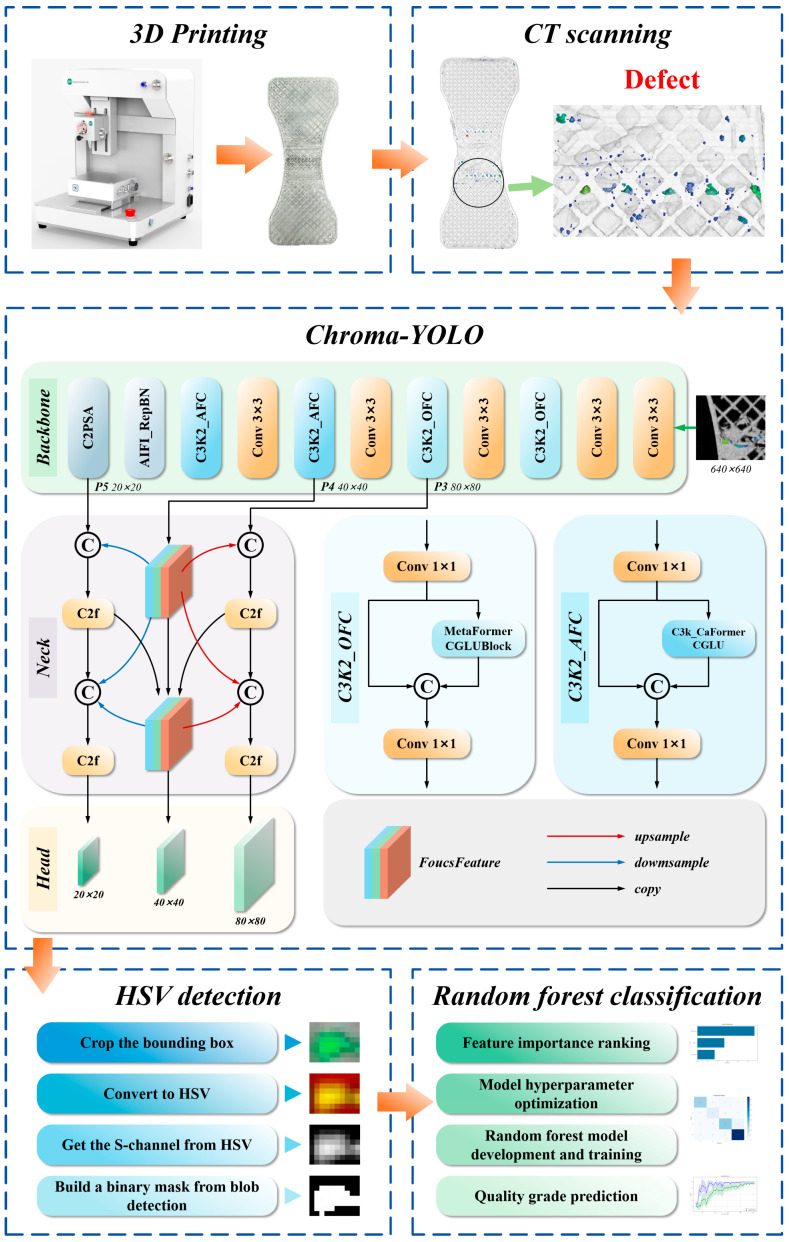
The framework flow: 3D Printing + CT scanning + Chroma-YOLO + HSV detection + Random prediction model.

To assess the mechanical properties and provide a baseline for fatigue testing, four PLA samples were subjected to monotonic tensile testing with an MTS Tytror 250 N axial machine (Mechanical Testing & Simulation, Dallas County, TX, USA) at a 0.5 mm/min strain rate. The geometry is shown in Figure 1, with the load applied axially. Mechanical parameters were averaged from four tests, as per the Chinese standard GB/T 1040.1-2018 [28].

Radial defect characterization of the 18 PLA samples was performed with a Nanotom 160 CT system (GE, Boston, MA, USA), configured at a 500 mm source–detector distance, 300 mm source–sample distance, and a resolution of 30 μm. All CT projections were reconstructed volumetrically with the Datos algorithm, and three-dimensional visualization of defects was subsequently achieved using Vgstudio Max 3.1.

Following CT-based internal defect characterization, fatigue experiments were performed under controlled laboratory conditions to evaluate the mechanical endurance of the 3D-printed PLA specimens. All tests were conducted at room temperature using an MTS Tytror (Houston, TX, USA) 250 N axial loading system equipped with a precision servo-hydraulic actuator. The experiments adopted a load-controlled sinusoidal cyclic loading mode, with a maximum applied load of 30 N, a stress ratio of R = 0 (tensile–compressive asymmetry), and a cyclic frequency of 20 Hz. Each specimen was subjected to continuous cyclic loading until either fracture occurred or the number of cycles reached 1 × 10^6^, which was defined as the fatigue endurance limit. Specimens that survived beyond this threshold without visible failure were considered to have achieved fatigue resistance, indicating that the applied stress amplitude was below the material’s critical fatigue threshold. During testing, the surface and fracture behavior were continuously monitored to correlate defect characteristics with fatigue life outcomes [29].

### 2.2. Chroma-YOLO

#### 2.2.1. YOLOv11

Redmon et al. redefined object detection as a regression problem through the YOLO algorithm, which completes detection in a single forward pass via a convolutional neural network, significantly simplifying the detection process and improving processing speed [30]. Since YOLO-v1’s initial release in 2015 up to YOLO-v8 in 2023, the YOLO series quickly became mainstream in the computer vision field due to its efficient single-stage architecture [31]. YOLOv11, released in 2024 as a representative single-stage detection model, reduced parameters by 22% while increasing mAP accuracy through innovative module design. This series of technical evolutions has demonstrated significant application potential in fields such as medical imaging and industrial inspection. YOLOv11 strengthens robustness by introducing novel modifications to both its backbone and detection head, while incorporating multi-scale feature fusion together with attention mechanisms. It integrates convolution with self-attention mechanisms to enhance recognition of complex details and small objects. The architecture consists of three primary modules: the input layer, backbone network, and detection head. Its backbone adopts a hierarchical structure for extracting features, and the neck integrates multi-resolution feature maps via upsampling and concatenation, where C3k2 modules are applied to reinforce feature representation. The detection head improves recognition capacity using a structured multi-scale strategy, producing predictions concurrently at three different scales (P3, P4, and P5). Such a parallel multi-scale design allows efficient detection of targets across different size ranges. YOLOv11 also optimizes its loss function, specifically by introducing refined IoU-based losses for bounding box regression, demonstrating exceptional adaptability and precision in specialized tasks such as 3D printing defect detection [32].

Inspired by YOLOv11, this research proposes Chroma-YOLO, an enhanced YOLOv11n-based model that integrates the AttForm attention enhancement network, TriFusion feature fusion architecture, RepFocus self-attention reparameterization module, and BIoU boundary penalty loss function module. This model detects internal defects in polylactic acid specimens. Subsequently, an HSV-based color separation method combined with a random forest algorithm identifies internal defects and predicts fatigue life. The overall process is illustrated in Figure 1.

#### 2.2.2. AttForm Attention Enhancement Network

Although the traditional C3k2 module (CSP Bottleneck with 3 × 3 Convolution) performs well in some visual tasks, it still faces certain limitations when processing images with complex structures [33]. The neck structure of YOLOv11 fuses multi-resolution feature maps through upsampling and concatenation, enhancing feature representation using improved C3k2 modules. This approach addresses the limitations of traditional convolutions in capturing long-range dependencies and reduces computational complexity, enabling the model to efficiently handle detection tasks for objects of different sizes simultaneously [34].

**Figure 2:**
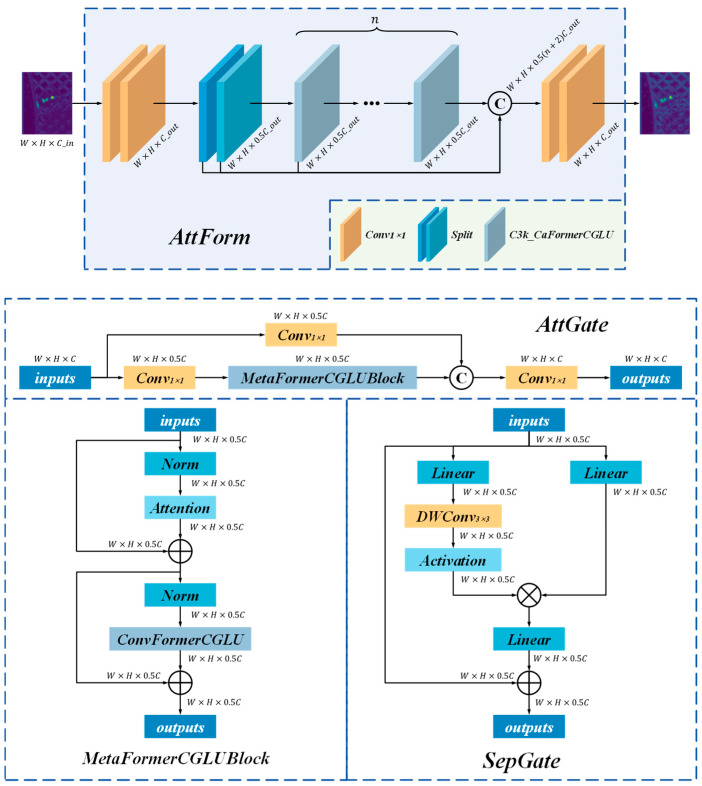
Process flow diagram of the AttForm module.

The AttForm attention enhancement module, constructed on a hybrid attention mechanism, integrates two components: the SepGate unit and the AttGate unit., as illustrated in Figure 2. The feature extraction module successfully integrates the local feature extraction capability of CNNs with the global modeling advantages of Transformers, providing an effective and high-performance framework for internal defect detection in polylactic acid specimens. These modules are designed upon the MetaFormer framework, where SepConv-based separable convolution and MF_Attention serve as primary feature mixers, with Convolutional Gated Linear Units (CGLU) further boosting feature representation. By combining the local extraction capability of CNNs with the global modeling power of Transformers, this design substantially improves accuracy in defect recognition under complex backgrounds, while keeping computation efficient.

The SepGate module, based on the MetaFormer framework, constructs an efficient feature extraction structure by combining SepConv with convolutional gated linear units (CGLU). This module approximates the global modeling capability of attention mechanisms by using depth-wise separable convolution, reducing computational complexity, and dynamically modulating feature responses through a gating mechanism. In the mathematical expression of this module, considering an input feature tensor $X\in {\mathbb{R}}^{W\;\times \;H\;\times \;C\_in}$, in which B denotes the batch size, C indicates the number of input channels, H and W represent the feature map’s height and width, respectively. The module first projects the input through a 1 × 1 convolution and splits it into two feature paths. In the feature extraction path, the SepConv operation implements feature transformation through a series of pointwise and depth wise convolutions. For an input X, SepConv can be represented as a continuous transformation sequence, as shown in Equation (1).

(1) $$Z={\mathrm{PW}}_{2}(\sigma_{2}(\mathrm{DW}(\sigma_{1}({\mathrm{PW}}_{1}(X)))))$$

where W_1_ represents a linear projection with a parameter matrix of size $W_{i}\in {\mathbb{R}}^{C\times C_{\mathrm{med}}}$, DW denotes a depthwise convolution operation with kernel size k, and $\sigma_{i}$ represents a non-linear activation function. This decomposition reduces the computational complexity from $O\left(k^{2}C^{2}\right)$ of standard convolution to $O\left(k^{2}C+C^{2}\right)$, while maintaining effective feature extraction capabilities.

In the MetaFormerCGLUBlock, the SepConv module is integrated as a specialized token mixer, enhancing feature expression capability through layer normalization (LN) and residual connections. Its computational process follows the principle of normalize-first then mix, as shown in Equation (2),

(2) $$X^{\prime }\;=\;X\;+\;\alpha_{1}\cdot D_{1}\left(\mathrm{SepConv}({\mathrm{LN}}_{1}(X))\right)$$

where $\alpha_{1}$ is a learnable scale factor, and *D*_1_ represents the Drop Path operation, thereby strengthening the generalization performance of the model.

The CGLU module improves feature representation by incorporating a gating strategy that adaptively adjusts channel significance. This gating process produces signals that either emphasize or attenuate particular feature channels according to the input, thereby facilitating the model’s capacity to capture complex patterns. Given input features X, the CGLU computation process is shown in Equation (3):

(3) $$Y_{\mathrm{CGLU}}=X+{\mathrm{Conv}}_{1\times 1}\left(\mathrm{DWConv}(X_{a})⊙X_{b}\right)$$

where ⊙ represents the Hadamard product, and DWConv is a depth wise convolution with non-linear activation. This gating mechanism can adaptively suppress irrelevant features while enhancing the representation of key defect areas.

The entire SepGate computation process can be represented as a cascade of feature transformation and fusion operations, achieving multi-path information transmission through the CSP (Cross Stage Partial) structure and combining the advantages of the MetaFormer architecture and convolutional operations, as expressed in Equation (4),

(4) $$Y={\mathrm{Conv}}_{1\times 1}\left(\left[X_{1},X_{2},M_{1}(X_{2}),M_{2}(X_{2}),...,M_{n}(X_{2})\right]\right)$$

where M_i_ represents the i-th MetaFormerCGLUBlock. This design efficiently extracts and fuses multi-branch features, making it particularly suitable for capturing defect features of different scales and morphologies in polylactic acid specimens.

The MF_Attention mechanism implements dynamic association modeling between arbitrary positions on the feature map. For input features $X\in {\mathbb{R}}^{B\;\times \;H\;\times \;W\;\times \;C}$, The attention mechanism initially derives the query (Q), key (K), and value (V) representations via linear projection operations. Unlike traditional attention mechanisms, a multi-head design to capture information patterns in different subspaces in parallel, as shown in Equation (5),

(5) $$Q,K,V=\mathrm{Reshape}\left(\mathrm{Linear}(X)\right)\in {\mathbb{R}}^{B\times N_{h}\times L\times d_{h}}$$

where *N_h_* represents the number of attention heads, *L* is the sequence length, *d_h_* is the dimension of each head, satisfying $N_{h}\cdot d_{h}=C_{\mathrm{attn}}$. Each attention head independently calculates attention scores, as shown in Equation (6),

(6) $$A_{i}=\mathrm{softmax}\left(\frac{Q_{i}K_{i}^{⊤}}{\sqrt{d_{h}}}\right)\in {\mathbb{R}}^{B\times L\times L}$$

where A*_i_* represents the attention weight matrix for the i-th head, and scaling factor $\sqrt{d_{h}}$ is used to stabilize gradients. The attention output is calculated through weighted aggregation of value vectors:

(7) $$O_{i}=A_{i}V_{i}\in {\mathbb{R}}^{B\times L\times d_{h}}.$$

where Linear in Equation (8) refers to a linear transformation layer, and Concat represents the tensor concatenation operation that produces the final result by combining the outputs of all attention heads followed by linear projection.

(8) $$O=\mathrm{Linear}\left(\mathrm{Concat}\left[O_{1},O_{2},...,O_{N_{h}}\right]\right)\in {\mathbb{R}}^{B\times H\times W\times C}.$$

In the MetaFormerCGLUBlock, MF_Attention combines with CGLU to form a dual-path structure. The forward propagation process includes two consecutive residual blocks. This architecture allows the module to integrate the strengths of global attention with local convolution, thereby improving feature representation across spatial and channel dimensions. Through this mechanism, the model can adaptively concentrate on defect areas, while CGLU further filters and enhances discriminative features through its gating mechanism. This combination is particularly suitable for detecting defects in polylactic acid specimens with weak contrast or diverse morphological variations.

#### 2.2.3. TriFusion Feature Fusion Network

**Figure 3:**
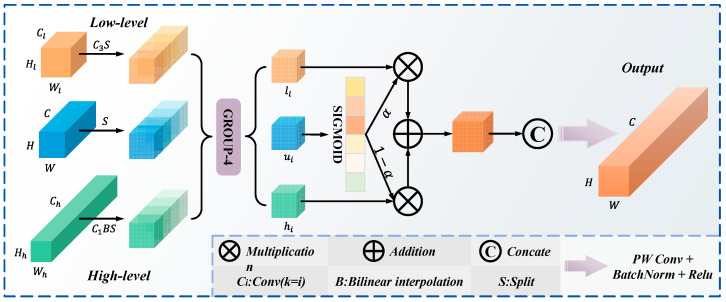
Construction process of the TriFusion module.

Feature pyramid models have limitations including the inability of simple feature concatenation to effectively represent complex defect information, single feature extraction paths struggling to adapt to diverse defect presentations, and the absence of adaptive fusion mechanisms, which prevent dynamic strategy adjustments based on image characteristics. These limitations reduce detection accuracy, particularly for small, blurred, or complex morphological defects [35]. To overcome these limitations, this study introduces a dynamic parallel feature network together with an adaptive fusion module. This method optimizes the fusion of features across different scales. The TriFusion module is designed based on multi-scale feature optimization theory, enabling adaptive collaboration between features of different scales through dynamic weighted fusion mechanism. The module consists of three key components: feature preprocessing, dynamic adaptive fusion, and residual enhancement, forming a complete feature optimization process. The module flow is shown in Figure 3. The feature preprocessing stage first unifies the dimensions of three input feature maps of different scales. For high-resolution features, X_low_ employs convolution transformation and bilinear interpolation operations; for low-resolution features X_high_, dilated convolution is applied; and for medium-resolution features X_mid_, adjustments are made through channels.

After preprocessing, the features are equally divided into four sub-feature blocks along the channel dimension: $X_{*}^{\prime }\;=\;[X_{*,i}^{\prime },X_{*,i}^{\prime },X_{*,i}^{\prime },X_{*,i}^{\prime }],*\in \{\mathrm{low},\mathrm{mid},\mathrm{high}\}$. Each sub-feature block undergoes adaptive fusion via the BAG module. The BAG module generates dynamic weight masks based on medium-resolution features: $\alpha_{i}=\sigma (X_{i}^{\prime }),i\in \{1,2,3,4\}$, where σ represents the Sigmoid activation function. The dynamic fusion process is represented as:

(9) $$Y_{i}=\alpha_{i}⊙X_{low,i}^{\prime }\;+\;\left(1\;-\;\alpha_{i}\right)⊙X_{\mathrm{high},i}^{\prime }.$$

where ⊙ represents the Hadamard product (element-wise multiplication). This soft attention-based weighted fusion mechanism enables the model to adaptively determine fusion weights at each spatial position, dynamically balancing detailed features and semantic features based on content complexity. The fused sub-features are recombined via channel concatenation, followed by channel dimension integration, the introduction of residual connections, and ultimately passed through batch normalization followed by a non-linear activation to generate the output.

The TriFusion module is constructed as a cascaded double-layer structure, forming a feature dynamic parallel network. The first layer processes multi-scale features P3/8, P4/16, and P5/32 extracted by the backbone, generating preliminary fusion features. The second layer further integrates the features generated by the first layer with additionally processed multi-scale features, forming enhanced features. This cascaded double-layer FDPN architecture establishes a bidirectional information flow network between features, enabling full interaction between features of different scales and greatly enhancing the network’s detection capability for various defect types.

#### 2.2.4. RepFocus Self-Attention Reparameterization Module

Deep neural networks often experience training instability and vanishing gradients in complex image processing tasks [33]. The traditional SPPF module in the YOLOv11 architecture has limitations including receptive field constraints, batch normalization offset, and imprecise spatial position encoding, especially when detecting defects in PLA specimens [36]. These issues are especially pronounced in scenarios involving highly reflective and low-contrast PLA materials [37], resulting in detection precision and recall rates inadequate for industrial applications [38].

**Figure 4:**
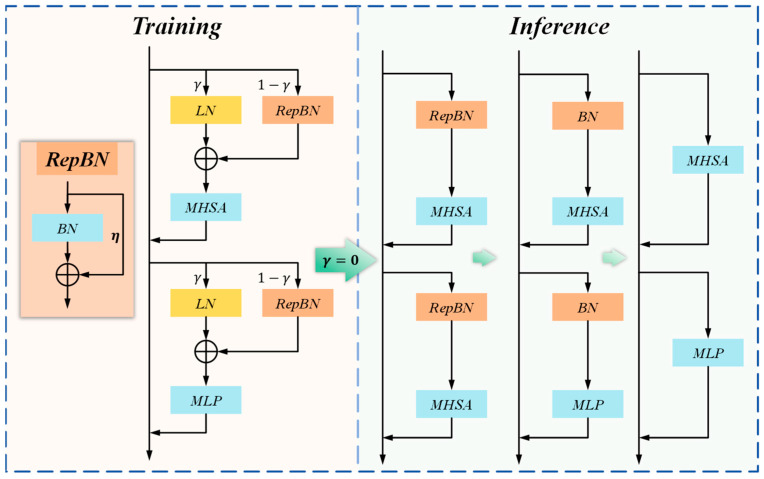
RepFocus hybrid normalization strategy.

This research proposes the RepFocus self-attention reparameterization module, which incorporates Transformer architecture and innovative normalization strategies to enhance defect discrimination, uses dynamic linear normalization to address distribution shifts between training and inference, and employs two-dimensional sine-cosine position encoding to preserve precise spatial information. Compared to traditional batch normalization methods, RepFocus adapts better to images of different sizes, improving training stability and detection accuracy. RepFocus captures long-range dependencies using self-attention mechanisms, enhancing sensitivity to small defects, especially in complex backgrounds. The normalization process of RepFocus, shown in Figure 4, illustrates a hybrid strategy combining layer normalization (LN) and reparameterizable batch normalization (RepBN). During training, parameter γ controls the weighting between the two normalization methods. The feature representations are simultaneously handled by multi-head self-attention (MHSA) and a multilayer perceptron (MLP), while RepBN internally integrates standard batch normalization (BN) with residual connections (parameter η). When γ is set to 0 during the inference phase, the model simplifies into three efficient architectures: RepBN-MHSA, BN-MHSA, or direct MHSA-MLP paths. This design optimizes inference efficiency and performance while maintaining training stability.

RepFocus is built upon a Transformer self-attention module, which measures positional similarity in the feature map and aggregates features through weighted summation, enabling each position to integrate information from other positions. The formula for computing self-attention is given by Equation (10):

(10) $$A\mathrm{ttention}(Q,K,V)=soft\max(\frac{QK^{T}}{\sqrt{d_{k}}})\cdot V$$

where Q, K, and V correspond to the Query, Key, and Value matrices, respectively, while d*_k_* denotes the dimensionality of the key vectors. Multi-head attention enables parallel learning of multiple attention patterns, capturing diverse relationships within images. In RepFocus, the input feature map passes through the self-attention module, which calculates its self-attention weights and generates an updated feature map via weighted summation. The updated feature map is then processed through a two-layer feed-forward neural network. The feed-forward network generally consists of two fully connected layers with a GELU activation function placed in between., defined as Equation (11):

(11) $$GELU(x)=0.5\cdot x\cdot (1+\tanh(\sqrt{\frac{2}{\pi }}\left(x+0.044715x^{3}\right))).$$

The RepFocus module employs residual connections and Layer Normalization (LN). Residual connections add the input feature map to the processed feature map, ensuring smooth information flow through the deep network [34].

#### 2.2.5. Boundary Penalty Loss Function Module

In object detection tasks, conventional IoU metrics evaluate solely the overlap between predicted and ground-truth boxes, while ignoring boundary cues and shape characteristics. This limitation becomes evident when the predicted box has minimal or no overlap with the ground truth box, the gradient approaches zero, severely affecting network learning efficiency. To address this issue, this research proposes the BIoU module, which constructs a more sensitive loss function by integrating boundary distance metrics with adaptive non-linear transformations, improving the model’s ability to recognize complex morphological defects in PLA specimens.

**Figure 5:**
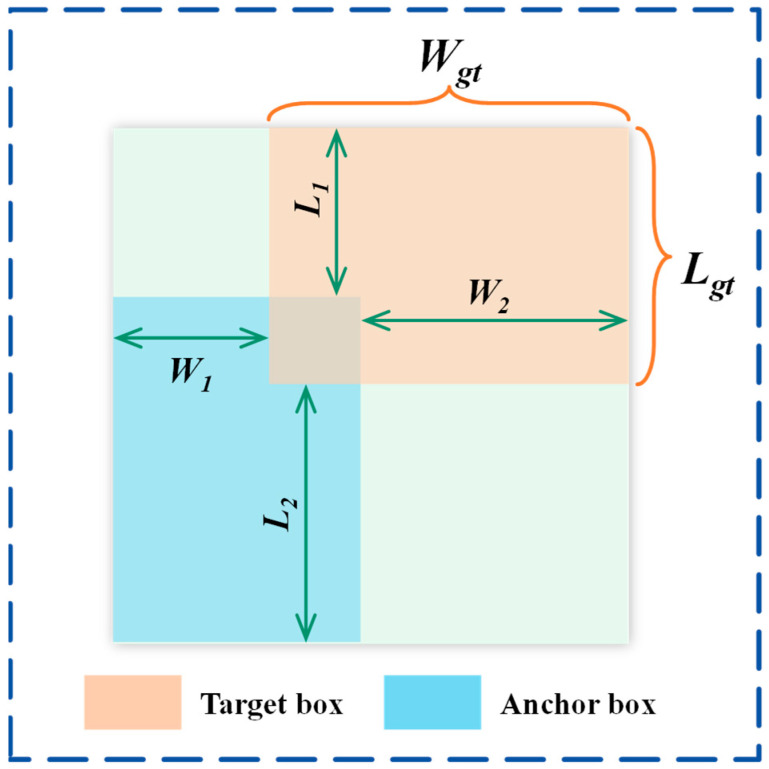
BIoU boundary penalty loss function module.

The BIoU module introduces a refined boundary spatial modeling method by calculating a normalized boundary distance factor P, which comprehensively describes the spatial relationship between predicted boxes and ground-truth targets. As shown in Figure 5, The boundary distance factor P is calculated by combining horizontal and vertical boundary differences, ensuring consistent sensitivity to defects of various scales. Its formula is given by Equation (12),

(12) $$P=\left(\frac{W_{1}}{W_{g}}+\frac{W_{2}}{W_{g}}+\frac{L_{1}}{L_{g}}+\frac{L_{2}}{L_{g}}\right)/4$$

where W and L represent the horizontal and vertical boundary distance between the predicted box and the ground truth box, and W_g_ and L_g_ denote the ground-truth box’s width and height. The core of the BIoU module is the introduction of an adaptive non-linear transformation mechanism that combines traditional IoU loss with a boundary-sensitive penalty term. Its formula is given by Equation (13):

(13) $$L_{PIoU}=L_{IoU}+1-e^{-P^{2}},0\leq L_{PIoU}\leq 2$$

When the predicted box perfectly matches the ground truth box boundaries, P approaches 0, $e^{-P^{2}}$ approaches 1, and the penalty term $1-e^{-P^{2}}$ approaches 0. In this case, L_PIoU_ is primarily determined by L_IoU_. However, as boundary differences increase, P increases, $e^{-P^{2}}$ decreases, and the penalty term increases, providing additional gradient for the loss function.

### 2.3. HSV Color Space-Based Defect Extraction

The HSV internal defect extraction module proposed in this paper precisely extracts defect features within polylactic acid materials from regions detected by Chroma-YOLO. This module leverages the unique characteristics of defect regions in HSV color space to achieve high-accuracy defect identification.

The defect detection workflow includes several steps: First, RGB images within the Chroma-YOLO detection frames are converted to HSV color space. This conversion effectively differentiates defect regions, since defects typically exhibit high saturation in HSV space, whereas defect-free regions appear as low-saturation gray areas. Direct extraction in RGB space results in lower detection precision and inaccurate defect area calculations. Second, the saturation (S) channel of the HSV image is extracted and normalized. Third, an adaptive threshold segmentation algorithm is applied to the normalized S channel to generate a binary mask. Finally, this mask is applied to the original image to extract defect regions. This workflow leverages the high-saturation characteristics of defects in HSV color space, simplifying the identification of defect regions against complex backgrounds. For clearer separation of defects from the background, the extracted saturation channel is normalized. The normalization process is described by Equation (14):

(14) $$S_{norm}\left(x,y\right)=255\times \frac{S\left(x,y\right)-S_{min}}{S_{max}-S_{min}}$$

where *S*(*x,y*) is the saturation value at coordinates (*x,y*), *S_max_* and *S_min_* are the maximum and minimum values of the *S* channel, respectively, and *S_norm_* is the normalized saturation value. When *S_max_* = *S_min_*_,_ the normalization result is set to 0 to avoid division by zero. The normalization maps saturation values to the range [0, 255], making defect regions visually prominent.

An adaptive threshold segmentation algorithm is applied to the normalized saturation channel to produce a binary mask. Given the variability of defect features, this research adopts an adaptive threshold strategy, where the threshold *T* determined by Equation (15):

(15) $$T=\alpha \cdot \mu_{S}+\beta \cdot \sigma_{S}$$

where μ_s_ and σ_s_ indicate the average value and standard deviation of the normalized saturation channel, respectively, and α and β are adjustment parameters. Through extensive experimental validation, α = 1.0 and β = 1.5 was found to achieve optimal results. The hyperparameters α and β were adjusted to balance detection precision and recall during model optimization. After multiple validation experiments, the configuration of α = 1.0 and β = 1.5 demonstrated the most stable convergence and highest mean average precision (mAP) among all tested combinations. This setting effectively suppressed overfitting while maintaining high sensitivity to small and low-contrast defects.

Finally, through bitwise operations, the binary mask is applied to the original RGB image to extract defect regions. If the original RGB image is denoted as *I_orig_* and the mask as *M*, the extracted defect region *I_defect_* expressed as:

(16) $$I_{defect}\left(x,y\right)=I_{orig}\left(x,y\right)\wedge M\left(x,y\right)$$

where ∧ represents the bitwise AND operation.

To further quantify defect severity, the defect area *A_defect_* is defined as the count of non-zero pixels, as shown in Equation (17):

(17) $$A_{defect}=\sum_{x,y}\delta \left(I_{defect}\left(x,y\right)\right)$$

where *δ*(*v*) = 1 if v ≠ 0, otherwise *δ*(*v*) = 0.

Through the described workflow, the proposed defect detection module effectively separates defect regions in polylactic acid materials from complex backgrounds, laying the foundation for subsequent defect classification and evaluation.

### 2.4. Random Forest Fatigue Life Prediction Model

After defect detection, this paper designs a defect prediction module based on the random forest algorithm to classify and evaluate the detected spot features [39]. The random forest algorithm is chosen for three key reasons: First, its ensemble learning characteristics reduce overfitting in individual decision trees; second, it is well-suited to processing non-linear relationships between features, such as the relationship between PLA material defect characteristics and fatigue life; and third, it provides feature importance assessment, aiding in understanding the contribution of defect parameters to life prediction [40].

This module uses supervised learning methods, comprehensively considering features such as defect quantity, maximum area, and total area to construct a high-precision defect prediction model. The workflow includes four main phases: feature extraction, data preprocessing, model training and optimization, and defect prediction.

The random forest enhances performance by generating several decision trees and aggregating their predictions. If the random forest consists of T decision trees, with the model of the t-th tree represented as *h_t_*(*x*), then the prediction function can be expressed as:

(18) $$H\left(X\right)=\frac{1}{T}\sum_{t=1}^{T}h_{t}\left(X\right).$$

To ensure diversity, the random forest introduces two types of randomness during training: bootstrap sampling and random feature selection. These randomness mechanisms reduce model variance, enhance generalization, and are well-suited for classification problems in high-dimensional feature spaces. To enhance model performance, this research systematically optimized random forest hyperparameters. The main hyperparameters are the number of decision trees T, the maximum number of features per node m, the tree depth d, and the minimum sample size for node splitting n_min. Hyperparameter optimization used grid search combined with cross-validation, with balanced accuracy as the objective function, as shown in Equation (19):

(19) $$BA=\frac{1}{K}\sum_{k=1}^{K}\frac{TP_{k}}{TP_{k}+FN_{k}}.$$

where *k* is the number of categories, and *TP_k_* and *FN_k_* are the true positive and false negative samples for category *k*, respectively.

## 3. Experiments and Results

### 3.1. Tensile, Fatigue, and SEM Results

**Figure 6:**
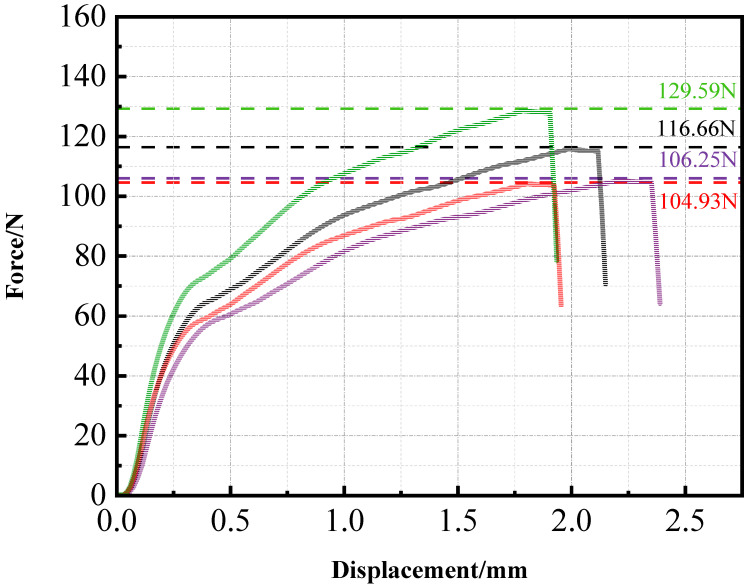
Force-displacement graph of the PLA specimen.

Tensile testing of PLA specimens was performed, and the static tensile curves for four sample groups are presented in Figure 6. The average results from the four tests were taken to characterize the mechanical properties of PLA. Because PLA is a brittle material, the specimens fractured quickly with limited deformation, making it difficult to accurately measure fracture strain. As a result, only the force–displacement curves could be obtained. The maximum loads borne by the four samples were 129.59 N, 116.66 N, 106.25 N, and 104.93 N.

**Figure 7:**
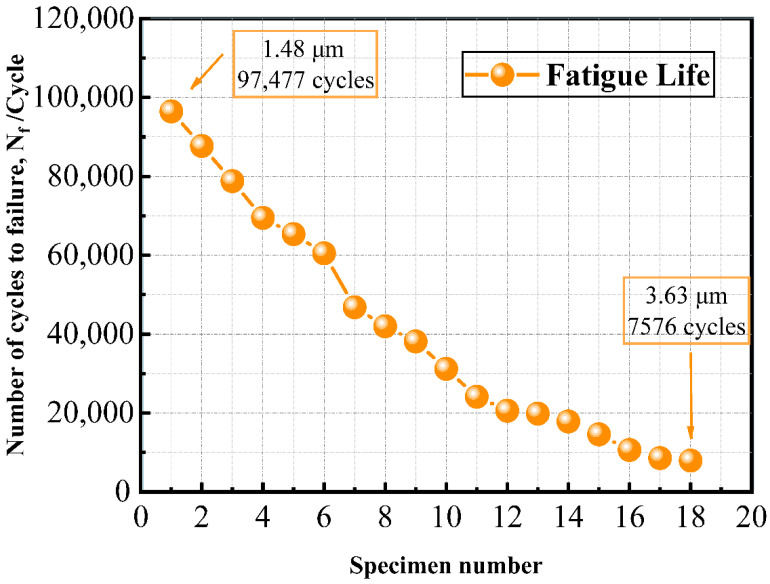
Number of cycles to fatigue life.

Defect size plays a crucial role in the fatigue performance of PLA specimens. The experiments used load control with 30 N, a stress ratio of 0, and a frequency of 20 Hz, with the fatigue limit defined at 10^6^ cycles. Arranged in descending order of fatigue life, the specimens exhibit lifespans ranging from 96,467 to 7576 cycles in Figure 7. Due to variations in the number and size of internal defects, their fatigue life shows considerable scatter.

**Figure 8:**
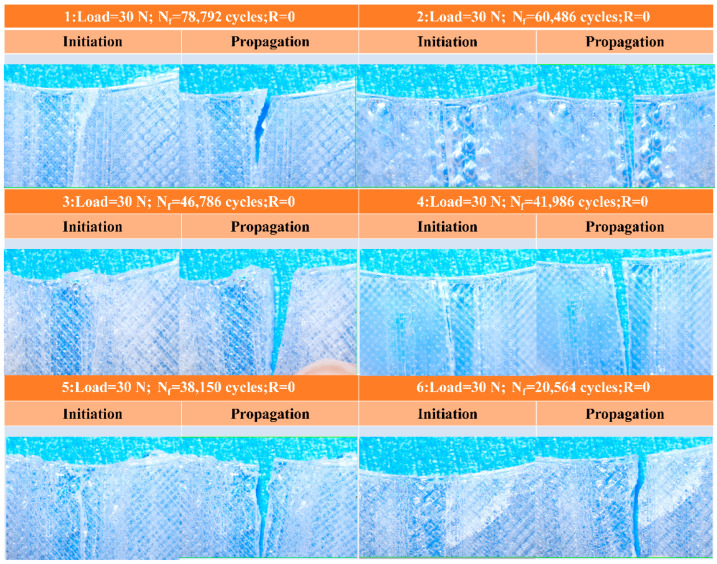
Fracture analysis of PLA specimens.

The fracture morphology of polylactic acid (PLA) was examined with an electron microscope to analyze the process of crack initiation and subsequent propagation. As illustrated in Figure 8, the initial cracks in PLA specimens typically form on planes nearly perpendicular to the direction of the cyclic axial load. This mode of initiation governs the following propagation phase, during which cracks advance along a trajectory also oriented perpendicular to the loading axis.

**Figure 9:**
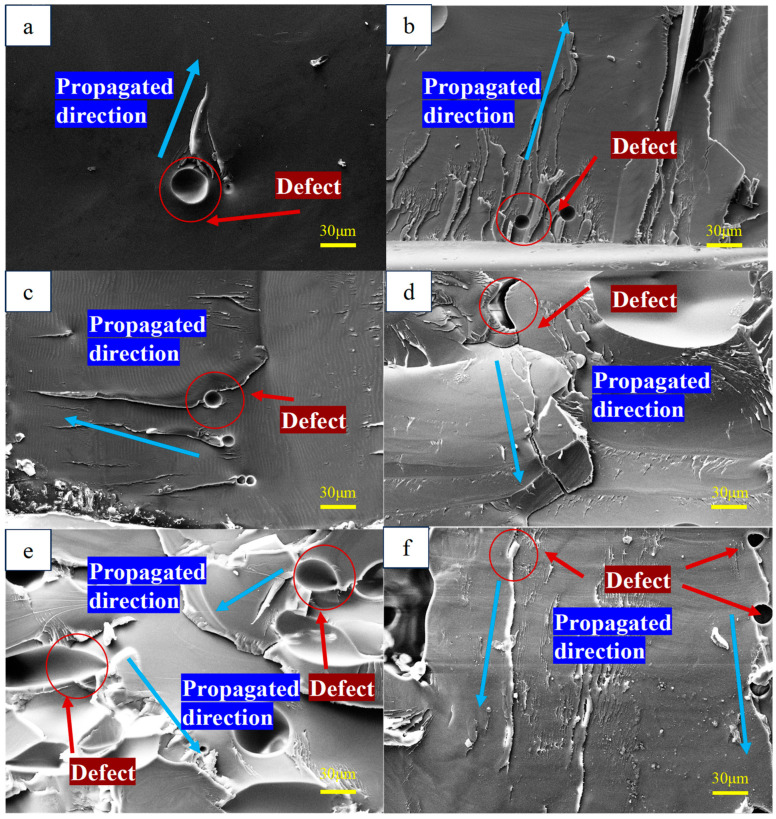
SEM results of PLA specimens. (**a**–**f**) different specimens.

The fracture morphology of PLA was examined using scanning electron microscopy to analyze crack initiation and propagation behavior. As shown in Figure 9, cracks consistently initiated on planes nearly orthogonal to the cyclic axial load direction. This initiation mode subsequently governed the propagation stage, where cracks extended along a path orthogonal to the applied force. Owing to the AM process, PLA filaments are deposited side by side to form planar layers, which are then stacked vertically to build the specimen. Fractures between adjacent filaments act as the primary sites for crack initiation. The localized fracture and shrinkage observed between neighboring filaments, accumulate across successive layers and ultimately result in fatigue failure.

### 3.2. Experimental Setup and Model Metrics

After obtaining CT images, layered slicing was applied on the CT images, and each specimen being sliced into 30 layers, resulting a total of 318 original images containing defect areas. Data augmentation was applied to the 318 original images through operations such as cropping, translation, brightness adjustment, noise addition, rotation, and mirroring, expanding the dataset to 3180 images. These images were divided into training and validation sets in a 7:3 ratio. The model was trained and validated in the Pytorch 2.1.0 framework. The experimental setup included a Windows OS, 32 GB of RAM, an Intel Core i7-14700 KF CPU, and an NVIDIA GeForce RTX 4070S GPU. Input images were resized to 640 × 640 pixels. The model was optimized using the Stochastic Gradient Descent (SGD) optimizer, with a batch size of 32 and 300 training epochs. During detection, both accuracy and speed were considered. Accuracy was measured using Precision, Recall, mAP at IoU 0.5 (mAP50), and mAP across IoU thresholds from 0.5 to 0.95 (mAP50-95).

1. Precision: Precision refers to the proportion of true positive samples among all samples predicted positive. Its formula is given by Equation (20):
  (20) $$\mathrm{Precision}=\frac{\mathrm{TP}}{\mathrm{TP}+\mathrm{FP}}$$
2. TP (True Positive) is the count of correctly detected targets, and FP (False Positive) is the count of incorrectly detected ones.
3. Recall: Recall refers to the proportion of correctly predicted positive samples among all actual positive samples. Its formula is given by Equation (21):
  (21) $$\mathrm{Recall}=\frac{\mathrm{TP}}{\mathrm{TP}+\mathrm{FN}}.$$
  where FN (False Negative) represents the number of targets that were not detected.
4. mAP50: mAP50 (Mean Average Precision at IoU threshold 0.5) represents the Average Precision when the IoU (Intersection over Union) threshold is set at 0.5. The formula for Average Precision is shown in Equation (22):
  (22) $$\mathrm{AP}=\int_{0}^{1}p\left(r\right)dr\approx \sum_{i=1}^{n}\left(r_{i+1}-r_{i}\right)\cdot p_{\mathrm{interp}}\left(r_{i+1}\right)$$
5. mAP50-95: mAP50-95 is the mean of Average Precision values across IoU thresholds from 0.5 to 0.95, with a step size of 0.05. Its formula is given by Equation (23):
  (23) $$\mathrm{mAP}50-95=\frac{1}{10}\sum_{t=0.5}^{0.95}{\mathrm{mAP}}_{t}$$
  where t represents the IoU thresholds from 0.5 to 0.95, with a step size of 0.05.

For model speed evaluation, Parameters and Giga Floating Point Operations Per Second (GFLOPS):

1. Parameters: Parameters refer to the total number of trainable parameters in the model. The total parameter count is typically expressed in millions (M) or billions (B).
2. Giga Floating Point Operations Per Second (GFLOPS): GFLOPS is a unit of measurement for assessing the processing capability of computing devices or algorithms.

### 3.3. Module Comparison Experiments

#### 3.3.1. AttForm Module Comparison Experiments

To verify the effectiveness of the proposed AttForm module in detecting internal defects in polylactic acid specimens, detailed comparisons were conducted with mainstream feature extraction modules, including C3k2-EMBC, C3k2-iRMB-DRB, C3k2-ConvFormer-CGLU, and C3k2-PoolingFormer-CGLU.The experimental results are presented in Table 1.

The experimental results demonstrate that the proposed AttForm module exhibits significant advantages across all performance metrics. Compared to the baseline model, AttForm improved the mAP50 metric by 7.6% (from 69.6% to 77.2%) and the mAP50-95 metric by 2.6% (from 24.1% to 26.7%). Notably, these improvements were achieved while reducing the parameter count by 17.9% (from 2.8 M to 2.3 M), demonstrating the efficiency of the model design. The AttForm module boosts feature extraction by using dual attention mechanisms in both channel and spatial dimensions. Its lightweight design reduces computational load, enabling faster processing while preserving detection accuracy.

#### 3.3.2. TriFusion Module Comparison Experiments

To validate the advantages of our proposed TriFusion module in the internal defect detection task of polylactic acid specimens, this module was compared with various mainstream object detection models, including FDPN, ContextGuideFPN, CGRFPN, and EMBSFPN. The relevant experimental results are presented in Table 2.

Analysis of the results reveals that TriFusion demonstrates significant advantages over other Neck structures. Compared to CGRFPN, TriFusion achieved a 6.6% higher Precision, 1.5% higher mAP50 metric, and 0.8% higher mAP50-95, while simultaneously reducing GFLOPS by 14.0% (from 8.6 to 7.4) and decreasing parameter count by 18.2% (from 3.3 M to 2.7 M). These results indicate that TriFusion effectively strengthens the model’s proficiency to extract defect features and improve localization accuracy through innovative feature fusion strategies while maintaining efficient computational complexity.

#### 3.3.3. RepFocus Module Comparison Experiments

The effectiveness of the RepFocus module for internal defect detection in PLA specimens was compared to several popular detection models, such as Focal Modulation, SPPF-LSKA, and AIFI. Experimental results are summarized in Table 3.

Analysis of the tabulated data shows that RepFocus exhibits comprehensive performance advantages compared to other models. Compared to FocalModulation, RepFocus achieved a 2.5% improvement in Precision, a 4.1% increase in mAP50 metric, and a significant 3.7% enhancement in the critical mAP50-95 metric, while reducing GFLOPS by 12.0% (from 7.5 to 6.6). These results indicate that by introducing reparameterizable batch normalization, RepFocus successfully improves the model’s ability to extract features and detect small targets detection precision, while improving computational efficiency, providing strong support for high-precision internal defect detection in polylactic acid specimens.

#### 3.3.4. BIoU Loss Function Comparison Experiments

To verify the effectiveness of our proposed BIoU model in the internal defect detection of polylactic acid specimens, this model was compared with several mainstream loss function models, as shown in the table. Table 4 lists experimental results under different modules, including Precision, Recall, and mAP metrics, aiming to comprehensively evaluate the performance of each model in this task.

Analysis of the tabulated data demonstrates that BIoU exhibits comprehensive advantages compared to other loss functions. Compared to wise_WIoU, BIoU improved the Precision by 2.8% and the Recall by 6.4%, while maintaining the same GFLOPS, with a slight increase in parameter count from 3.1 M to 3.2 M. These results demonstrate that Powerful-IoU addresses the deficiencies of traditional IoU loss in processing small defects in polylactic acid specimens by accurately modeling bounding box distance relationships, providing essential support for high-precision defect detection.

### 3.4. Ablation Experiments

Comprehensive ablation experiments were carried out to evaluate the Chroma-YOLO effectiveness in detecting internal defects in polylactic acid specimens, comparing performance against several mainstream object detection models. As shown in Table 5, the complete Chroma-YOLO model was progressively constructed, from individual module improvements to a combination of multiple modules. Compared to the baseline YOLOv11n model, the proposed Chroma-YOLO achieved improvements of 9.7% in Precision (from 71.5% to 81.2%), 12.5% in Recall (from 69.8% to 82.3%), 6.3% in mAP50 (from 76.7% to 83.6%), and 7.3% in mAP50-95 (from 27.2% to 34.5%). These improvements demonstrate the significant value of the proposed enhancements for internal defect detection in polylactic acid specimens.

**Figure 10:**
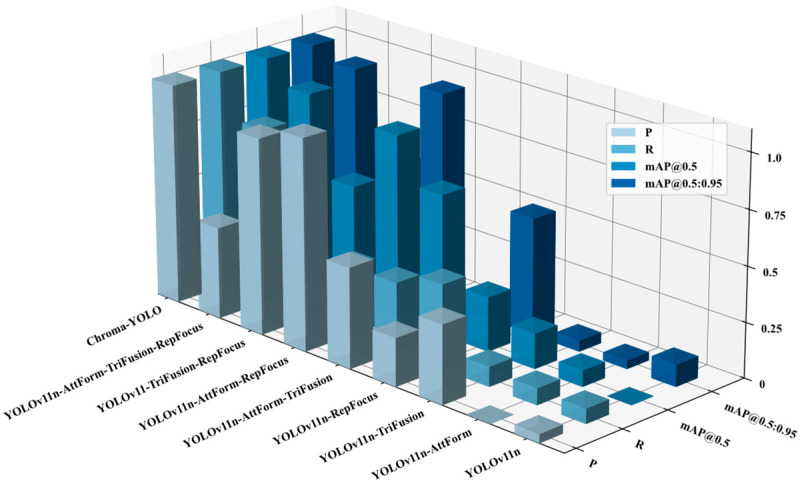
Ablation experiment results.

The ablation experiment results clearly demonstrate each module’s contribution to model performance. As shown in Figure 10, in single module improvements, AttForm, TriFusion, and RepFocus each exhibited enhancements in different performance metrics, with RepFocus achieving the most significant improvements in Recall and mAP50-95. Among dual-module combinations, the integration of AttForm with RepFocus yielded the best results, with Precision increasing to 80.8% and mAP50 rising to 82.5%, significantly outperforming other combinations. Ultimately, the complete Chroma-YOLO model, integrating all four modules, achieved optimal performance with significant improvements across all metrics. This result not only validates the rationality of each module’s design but also highlights their synergistic effect when combined, providing solid technical support for high-precision internal defect detection in polylactic acid specimens.

### 3.5. Comparative Experiments of Different Detection Models

The Chroma-YOLO model’s capability to detect internal defects in PLA specimens was assessed in comparison with leading object detection models, including YOLOv5n, YOLOv5s, YOLOv8, YOLOv10 variants, and the YOLOv11n baseline model. The experiment comprehensively assessed their performance in detection tasks by comparing various evaluation metrics, including GFLOPs, parameter count, precision, recall, mAP50, and mAP50-95. The experimental results are presented in Table 6.

**Figure 11:**
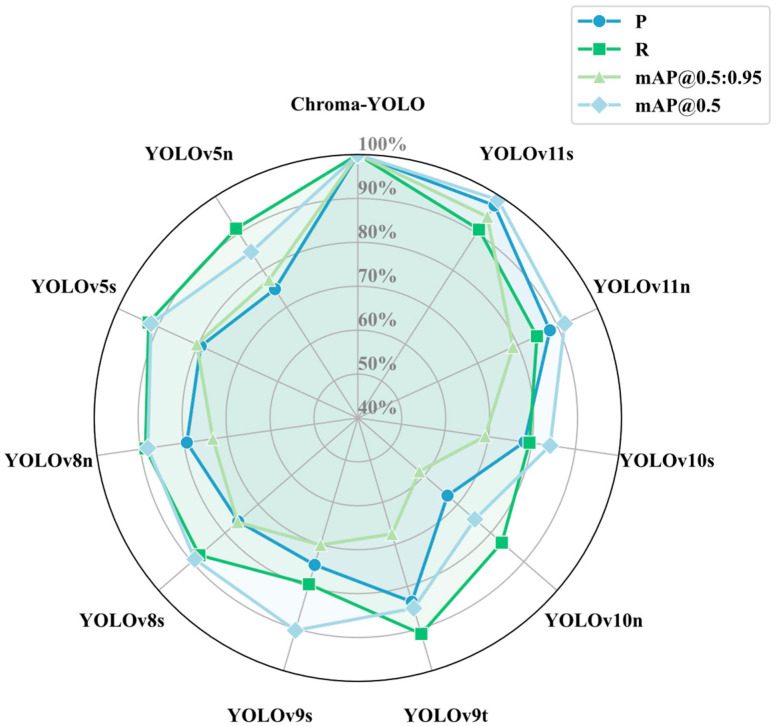
Comparison results of YOLO series models.

As illustrated in Figure 11, the Chroma-YOLO model demonstrated significant improvements across all evaluation metrics, particularly in key indicators such as Precision, Recall, and mAP. In terms of Precision, Chroma-YOLO achieved 81.2%, significantly higher than YOLOv11n’s 71.5% and YOLOv5s’s 64.4%. This improvement can be attributed to the AttForm and TriFusion modules introduced in Chroma-YOLO, which effectively enhanced feature extraction and information fusion capabilities, helping the model better capture and understand defect characteristics in polylactic acid specimens. Regarding Recall, Chroma-YOLO reached 82.3%, showing substantial improvement compared to YOLOv11n’s 69.8% and YOLOv5s’s 76%. This enhancement primarily benefited from the introduction of BIoU, which optimized the detection box regression process and improved the model’s ability in boundary box localization and defect area coverage, resulting in Chroma-YOLO’s superior recall performance.

**Figure 12:**
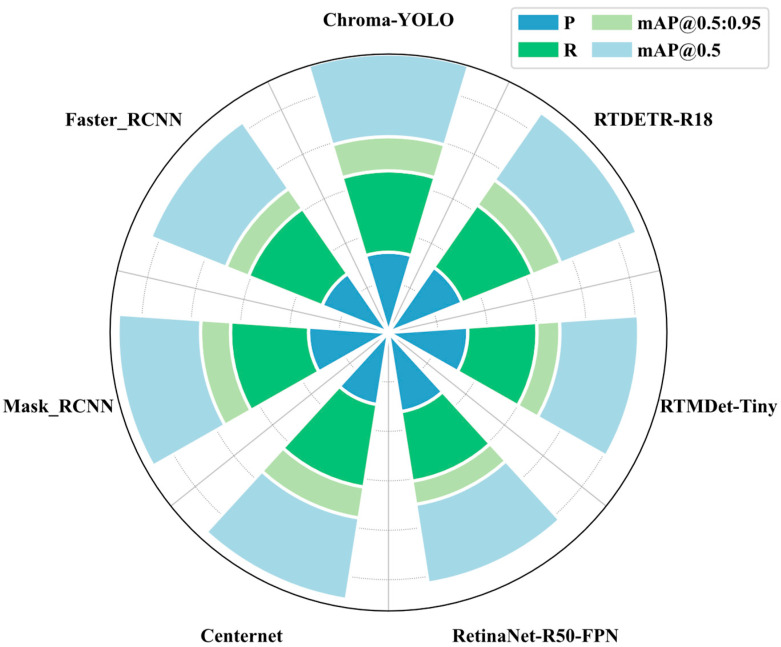
Comparative experimental results of different models.

As shown in Figure 12 and Table 6, Chroma-YOLO also exhibited significant advantages compared to other lightweight models. Compared to RetinaNet-R50-FPN, with similar computational complexity, Chroma-YOLO’s Precision was slightly higher by 0.1%, but its Recall substantially increased by 11%, mAP50 improved by 3.0%, and mAP50-95 increased by10.9%. Compared to RTMDet-Tiny, with similar computational complexity, Chroma-YOLO achieved significant improvements across all evaluation metrics, particularly with a 11.4% increase in the mAP50-95. Even when compared to the high-precision RTDETR-R18, Chroma-YOLO reduced GFLOPS by 86.8% and parameter count by 84.4%, while improving Precision, Recall, and mAP50 metrics by 0.9%, 5.4%, and 0.7%, respectively, with mAP50-95 increasing by 3.8%.

**Figure 13:**
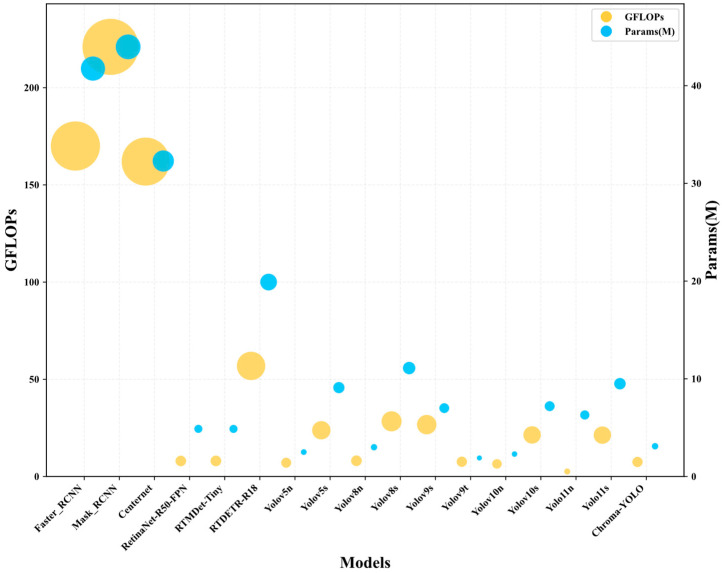
Comparison results of GFLOPS and Parameter.

As illustrated in Figure 13 and Table 6, the Chroma-YOLO model demonstrated superior balance between performance and efficiency compared to all models in the comparison. Compared to models with higher computational resource consumption (such as YOLOv5s, YOLOv8s, YOLOv9s, YOLOv10s, and YOLOv11s), Chroma-YOLO achieved higher performance metrics with significantly lower GFLOPS and parameter counts. For example, compared to YOLOv11s, Chroma-YOLO reduced GFLOPS by 64.8% (from 21.3 to 7.5) and parameter count by 67.4% (from 9.5 M to 3.1 M), while improving Precision by 2%, Recall by 7.4%, mAP50 by 0.8%, and mAP50-95 by 1.9%. Even compared to lightweight models of similar computational complexity (such as YOLOv5n, YOLOv8n, YOLOv9t), Chroma-YOLO outperformed better across all performance metrics; for instance, compared to YOLOv9t, Precision improved by 13.3% and Recall by 7.2%. Compared to traditional two-stage detectors Faster R-CNN and Mask R-CNN, Chroma-YOLO significantly reduced computational complexity, with GFLOPS of only 7.5, representing reductions of 95.6% and 96.6%, respectively, and parameter count of only 3.1 M, reductions of 92.6% and 92.9%, respectively. These outcomes validate the effectiveness and efficiency of the Chroma-YOLO model for detecting internal defects in polylactic acid specimens, achieving an excellent balance between speed and precision.

**Figure 14:**
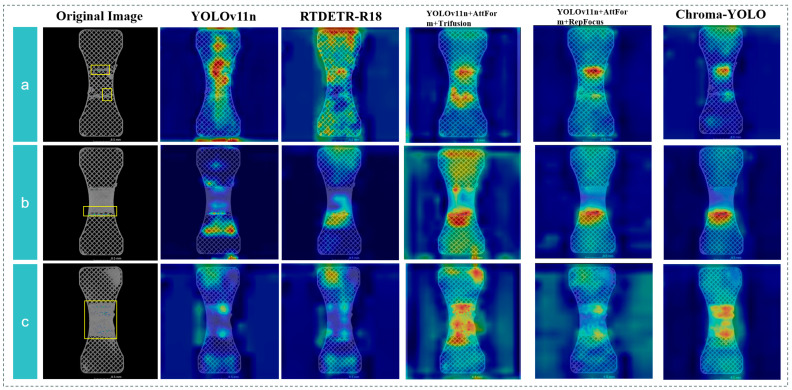
(**a**) Comparison of three models on different distributions; (**b**) Comparison of three models on sizes; (**c**) Comparison of three models on different quantities.

To thoroughly compare the perceptual capability of the Chroma-YOLO model in detecting internal defects in PLA specimens, the detection performance of two models were contrasted on defect images with different distributions, sizes, and quantities. The heatmaps shown in Figure 14 clearly present the attention distribution of different models on defect regions, intuitively demonstrating each algorithm’s focal localization capability in feature recognition, providing visual evidence for model performance evaluation. Compared to the basic YOLOv11n, the Chroma-YOLO model, integrating the AttForm, the TriFusion, and the RepFocus, significantly enhances the perception of defect regions. The introduction of the AttForm module expands the perceptual range, enabling more comprehensive capture of contextual information around defects. The TriFusion module provides significant advantages in multi-scale feature fusion, while the RepFocus module more precisely focuses on key points of defects, resulting in refined local feature representation.

The Chroma-YOLO model, through the effective combination of these three complementary modules, demonstrates more precise localization of defect regions in the heatmap, manifested as more focused hotspot areas with deeper colors. Compared to RTDETR-18, Chroma-YOLO exhibits superior defect perception capabilities while maintaining lower computational complexity, particularly for small and complex-shaped defects in polylactic acid specimens. This improvement addresses challenges in spatial state distribution, multi-scale detection, and multi-target identification which were previously susceptible to grid interference in unimproved models.

### 3.6. Defect Extraction in Polylactic Acid Specimens

**Figure 15:**
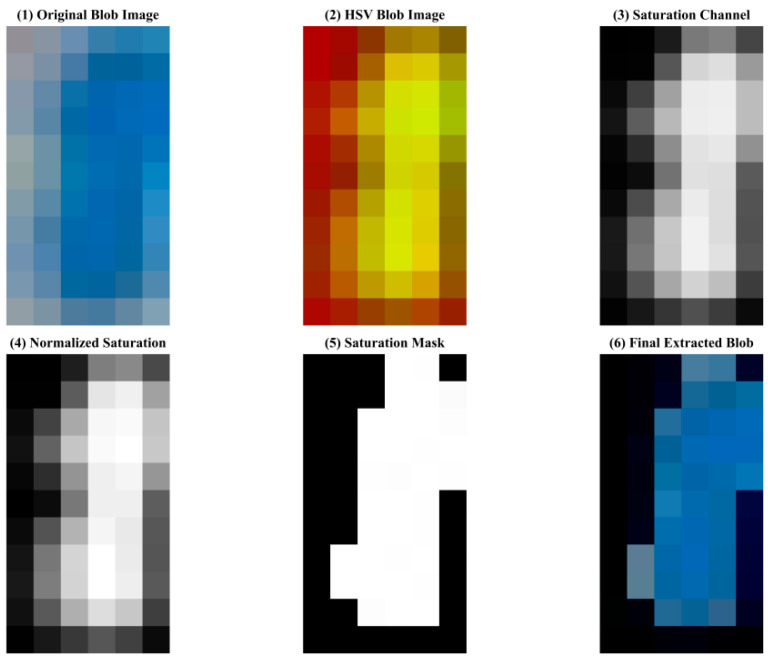
HSV defect extraction process.

The complete workflow for saturation channel-based defect extraction in internal defects of polylactic acid specimens is shown in the figure, sequentially displaying the original blob image, HSV-converted image, saturation channel, normalized saturation image, generated saturation mask, and finally extracted blob. This process is shown in Figure 15.

The HSV color space-based blob detection method effectively detects internal defects in polylactic acid specimens. After HSV conversion, defect regions appear as highlighted features in the saturation channel; normalization processing further enhances the contrast between defects and background; the saturation mask accurately marks defect contours; and the final extracted blob preserves the morphological and color characteristics of the original defect, providing high-quality detection results for subsequent defect classification and localization.

### 3.7. Fatigue Life Prediction for PLA Specimens

The fatigue performance of additively manufactured components is primarily influenced by the geometric characteristics of defects, including size position, morphology, and orientation. Among these, size has the greatest impact on fatigue behavior [38], followed by position [41], with morphology has a relatively minor influence. In the authors’ previous research [29], the fatigue limit of polylactic acid (PLA) specimens was predicted by considering the maximum size of internal defects using an improved Murakami equation. This article extends this work by incorporating three factors to predict the fatigue life of PLA specimens: defect quantity (N), maximum defect area (S_max_), and total defect area (S). Feature importance analysis of these three factors reveals that maximum defect area contributes most significantly to fatigue life prediction (importance of 0.483), followed by total defect area (0.371) and defect quantity (0.146).

To ensure model accuracy, 318 original images without enhancement were used for prediction. The dataset was divided into training (128 images), validation (110 images), and testing (80 images) sets. Since these images were derived from two-dimensional planes obtained by sectioning three-dimensional CT reconstructions of 18 specimens, and each specimen corresponding to only one fatigue life value, multiple images shared identical fatigue life measurements. To conduct objective and effective performance evaluation on independent data, the specimen fatigue data were stratified into four distinct categories, with predictive accuracy across these categories used as the key metric to assess the effectiveness of the fatigue life prediction model. The classification criteria for fatigue life prediction categories and the corresponding dataset distribution are presented in Table 7. Among them, the P1 level corresponds to the fatigue life of the specimen in the range of 0–25,000 cycles, P2 level corresponds to 25,000–50,000 cycles, P3 level corresponds to 50,000–75,000 cycles, and P4 level corresponds to 75,000–100,000 cycles.

This research employed the GridSearchCV method combined with cross-validation to systematically optimize random forest hyperparameters. The optimization strategy aimed to determine the ideal parameter values, including the number of decision trees (n_estimators), the maximum depth (max_depth), as well as the minimum samples needed for node splitting (min_samples_split) and leaf formation (min_samples_leaf). The hyperparameter grid is presented in Table 8, with the optimal hyperparameter combination identified as the BEST group. The selection of optimal parameters, including the learning rate, batch size, and HSV extraction thresholds, was based on iterative validation experiments. The learning rate was adjusted to ensure stable convergence during training, while the batch size was optimized to balance computational efficiency and model accuracy. The HSV threshold values were determined by comparing segmentation performance across multiple trials, ensuring accurate extraction of defect regions without introducing noise.

**Figure 16:**
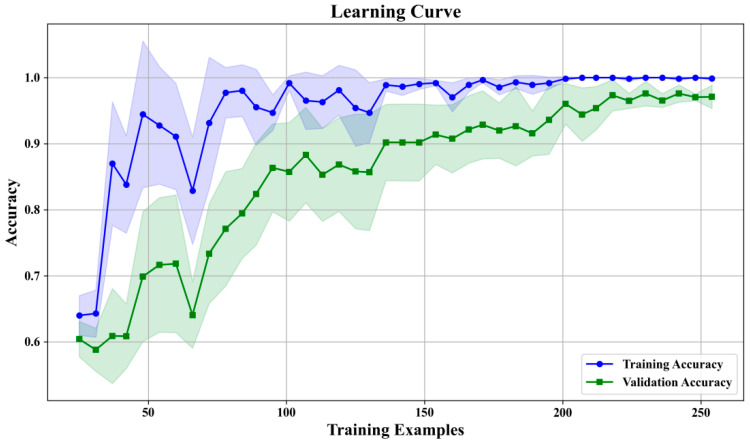
Random forest training process curves.

Through grid search, this research determined the optimal hyperparameter combination, with the number of decision trees = 26, maximum tree depth = NONE, minimum number of samples required for leaf nodes = 1, and minimum number of samples required for node splitting = 3. These parameter settings achieved high classification accuracy and strong generalization capability while maintaining moderate model complexity. The training process curve is shown in Figure 16.

Learning curve analysis indicates that the random forest prediction model demonstrated excellent convergence characteristics and generalization capability, stabilizing after reaching 200 training samples. Training accuracy remained in the 0.98–1.0 range and validation accuracy at 0.95–0.97 level, indicating a moderate gap. This result demonstrates not only the high precision and reliability of the random forest prediction system based on the Chroma-YOLO defect detection framework but also its significant engineering value for life management of polylactic acid materials.

**Figure 17:**
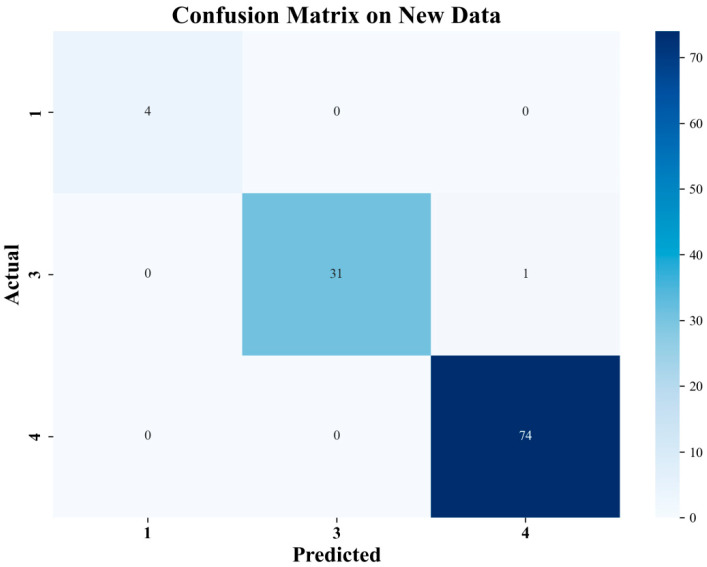
Validation set confusion matrix.

**Figure 18:**
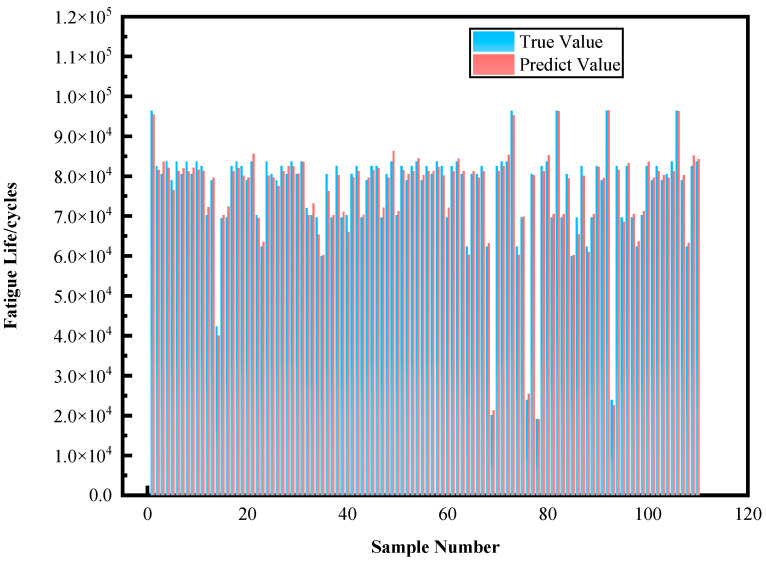
Validation set prediction results.

To verify the model’s generalization capability, this research conducted validation testing using a newly collected dataset of polylactic acid specimens. As shown in Figure 17, the confusion matrix and prediction results on the validation dataset demonstrate the model’s prediction performance across different fatigue life categories. The results show that P1 level and P4 level samples were all correctly predicted; among P3 level samples, 31 were correctly classified, with only 1 incorrectly predicted as P4 class. Overall, the validation dataset indicates that the proposed random forest model possesses excellent generalization capability, maintaining high prediction accuracy when processing new samples. The prediction results on the validation set are shown in Figure 18.

Analysis of the validation results shows that the model achieved an overall accuracy of 99.09% on new data, further exceeding the test set performance. Notably, the model performed exceptionally well in processing P4 level samples, achieving 100% prediction accuracy. Meanwhile, although slight confusion still exists between P3 and P4 levels, the model demonstrates excellent overall classification capability, fully proving the practicality and reliability of the random forest life prediction model trained on Chroma-YOLO defect detection results.

**Figure 19:**
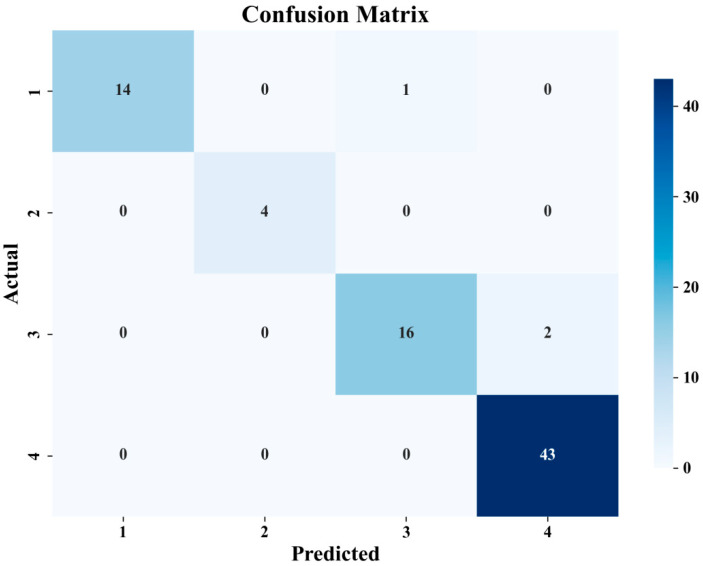
Confusion matrix for test set.

**Figure 20:**
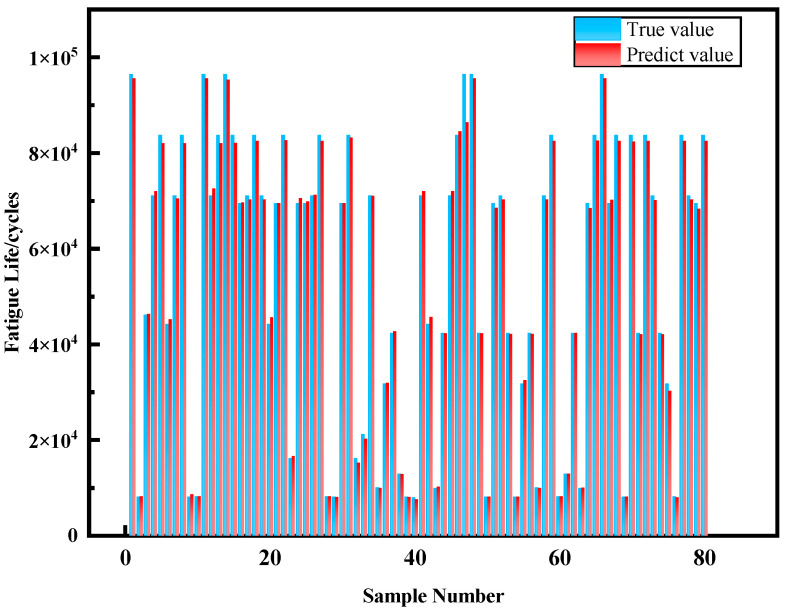
Test set and prediction results.

As shown in Figure 19, the confusion matrix on the test set clearly displays prediction results across four fatigue life categories. For the P1 level, 14 samples were accurately predicted; for the P2 level, 4 samples were correctly classified; for the P3 level, 16 samples were accurately predicted with only 2 incorrectly classified as category 4; and the P4 level achieved the highest accuracy with all 43 samples correctly predicted. This indicates that the random forest model performs consistently across different fatigue life categories, with particularly high accuracy for specimens with extremely short and long lifespans. The prediction results on the Test set are shown in Figure 20.

In terms of classification performance, the model achieved an overall accuracy of 96.25% on the test set, with only 3 samples incorrectly classified. Notably, the model exhibited slight confusion between P3 and P4 levels, possibly due to similarities in certain defect characteristics between these two categories. However, prediction performance for P1 and P2 lifespan samples was excellent, with no cross-misclassification occurring. These results demonstrate that defect features obtained through Chroma-YOLO detection effectively support the random forest algorithm in predicting fatigue life of polylactic acid specimens.

## 4. Conclusions

This research introduces a Chroma-YOLO Enhanced Integrated Framework for polylactic acid specimens based on the improved YOLOv11n. The integrated system establishes a complete defect detection to fatigue life prediction chain, mapping internal defect characteristics to fatigue life, providing an intelligent solution for polylactic acid quality control and reliability assessment. The main findings are as follows:

1. An integrated detection and prediction framework consisting of three major modules: Chroma-YOLO with four core innovations (AttForm attention enhancement network, TriFusion feature fusion architecture, RepFocus self-attention reparameterization module, and BIoU boundary penalty loss function), an HSV defect extraction module, and a random forest fatigue life prediction model.
2. Experimental results show that Chroma-YOLO outperforms the baseline YOLOv11n by improving Precision by 9.7% (71.5% to 81.2%), Recall by 12.5% (69.8% to 82.3%), mAP@50 by 6.9% (76.7% to 83.6%), and mAP@50-95 by 7.3% (27.2% to 34.5%).
3. The HSV defect detection module increases the contrast between defect regions and the background, significantly improving visibility. The random forest model achieves an accuracy of 96.25% on the test set and 99.09% on the validation set.

Despite the promising results, the proposed approach is limited by the dependence on CT image quality and its current validation on PLA materials only. Future work will focus on extending the method to multi-material systems and 3D reconstruction analysis to further improve its generality and practical applicability.

## Figures and Tables

**Table 1:** Results of AttForm module comparison experiments.

Model	GFLOPS	Params (M)	Precision (%)	Recall (%)	mAP50 (%)	mAP50-95 (%)
C3k2-EMBC	6.1	2.8	67.1	76.9	69.6	24.1
C3k2-iRMB-DRB	6.2	2.4	66.5	75.7	66.0	23.1
C3k2-PoolingFormer-CGLU	5.5	2.3	70.3	74.5	76.8	25.6
AttForm	6.0	2.3	71.1	69.9	77.2	26.7

**Table 2:** Results of neck comparison experiments.

Model	GFLOPS	Params (M)	Precision (%)	Recall (%)	mAP50 (%)	mAP50-95 (%)
FDPN	7.6	2.7	67.4	74.5	72.4	24.8
CGRFPN	8.6	3.3	68.1	75.3	76.3	26.0
EMBSFPN	6.5	2.12	65.5	75.4	77.3	24.8
TriFusion	7.4	2.7	74.7	70.1	77.8	26.8

**Table 3:** Results of RepFocus module comparison experiments.

Model	GFLOPS	Params (M)	Precision (%)	Recall (%)	mAP50 (%)	mAP50-95 (%)
FocalModulation	7.5	2.8	70.8	76.3	74.3	27.0
SPPF-LSKA	7.6	2.8	70.6	75.2	74.1	27.2
AIFI	7.5	3.15	66.2	77.8	74.1	25.1
RepFocus	6.6	3.2	73.3	74.0	78.4	30.7

**Table 4:** Comparison experiments of BIoU loss function.

Model	GFLOPS	Params (M)	Precision (%)	Recall (%)	mAP50 (%)	mAP50-95 (%)
Inner shape iou	7.5	3.2	81.6	63.6	77.5	31.7
wise_WIoU	7.5	3.1	70.3	75.8	79.8	33.7
Inner-Powerful-IoU	7.5	3.2	81.8	70.7	76.1	26.2
BIoU	7.5	3.2	73.1	82.2	83.6	34.2

**Table 5:** Ablation experiment results.

Model	GFLOPS	Params (M)	Precision (%)	Recall (%)	mAP50 (%)	mAP50-95 (%)
YOLOv11n	2.6	6.3	71.5	69.8	76.7	27.2
YOLOv11n-AttForm	6.0	2.3	71.1	69.9	77.2	26.7
YOLOv11n-Trifusion	7.4	2.7	74.7	70.1	77.8	26.8
YOLOv11n-RepFocus	6.6	3.2	73.3	74.0	78.4	30.7
YOLOv11n-AttForm-Trifusion	7.2	2.5	75.7	73.1	81.1	26.4
YOLOv11n-AttForm-RepFocus	6.3	2.9	80.8	73.1	82.5	34.2
YOLOv11n-TriFusion-RepFocus	7.7	3.3	80.1	68.9	80.4	31.0
YOLOv11n-AttForm-Trifusion-RepFocus	7.5	3.1	75.3	79.6	82.9	34.1
Chroma-YOLO	7.5	3.1	81.2	82.3	83.6	34.5

**Table 6:** Comparative experiment results.

Model	GFLOPS	Params (M)	Precision (%)	Recall (%)	mAP50 (%)	mAP50-95 (%)
Yolov5n	7.1	2.5	60.8	75.1	71	26.7
yolov5s	23.8	9.1	64.4	76.0	76.7	27.7
Yolov8n	8.1	3.0	64.4	73.1	73.8	25.3
Yolov8s	28.4	11.1	61.7	72.2	74.5	26.3
Yolov9s	26.7	7.0	60.8	65.4	75.6	24.2
Yolov9t	7.6	1.9	67.9	75.1	71.2	23.3
Yolov10n	6.5	2.3	54.4	68.6	62.9	20.2
Yolov10s	21.4	7.2	63.6	65.4	70.4	23.9
Yolov11n	6.3	2.6	71.5	69.8	76.7	27.2
Yolov11s	21.3	9.5	79.2	74.9	82.8	32.6
Faster_RCNN	170.0	41.75	72.5	80.2	81.9	24.5
Mask_RCNN	221.0	43.97	80.9	79.0	83.5	30.2
Centernet	162.0	32.29	73.8	84.6	83.3	31.6
RetinaNet-R50-FPN	8.0	4.88	81.1	71.3	80.6	23.6
RTMDet-Tiny	8.03	4.873	80.2	70.1	79.6	23.1
RTDETR-R18	56.9	19.9	80.3	76.9	82.9	30.7
Chroma-YOLO	7.5	3.1	81.2	82.3	83.6	34.5

**Table 7:** Fatigue life classification and category distribution.

Categories	N	S (pixel)	S_max_ (pixel)	Training Set	Validation Set	Test Set
P1	N > 20	S > 300	S_max_ ≥ 150	45	4	15
P2	10 < N < 20	200 < S < 300	S_max_ < 150	17	0	4
P3	10 < N < 20	100 < S < 200	S_max_ < 150	48	32	18
P4	0 < N < 10	0 < S < 100	S_max_ < 150	208	74	43

**Table 8:** Random forest hyperparameter combinations.

	n_estimators	max_depth	min_samples_split	min_samples_leaf
1	22	NONE	1	1
2	24	10	2	2
3	26	20	3	3
4	28	30	4	4
BEST	26	NONE	1	3

## Data Availability

The original contributions presented in this study are included in the article. Further inquiries can be directed to the corresponding author.

## Abbreviations

The following abbreviations are used in this manuscript:

PLA	Polylactic Acid
